# Examining the Benefits of Greenness on Reducing Suicide Mortality Rate: A Global Ecological Study

**DOI:** 10.3389/fpubh.2022.902480

**Published:** 2022-07-05

**Authors:** Aji Kusumaning Asri, Hui-Ju Tsai, Pei-Yi Wong, Hsiao-Yun Lee, Wen-Chi Pan, Yue-Leon Guo, Chi-Shin Wu, Huey-Jen Su, Chih-Da Wu, John D. Spengler

**Affiliations:** ^1^Department of Geomatics, National Cheng Kung University, Tainan, Taiwan; ^2^Institute of Population Health Sciences, National Health Research Institutes, Miaoli County, Taiwan; ^3^Department of Environmental and Occupational Health, National Cheng Kung University, Tainan, Taiwan; ^4^Department of Leisure Industry and Health Promotion, National Taipei University of Nursing and Health Sciences, Taipei, Taiwan; ^5^Institute of Environmental and Occupational Health Sciences, National Yang Ming Chiao Tung University, Taipei, Taiwan; ^6^Department of Environmental and Occupational Medicine, National Taiwan University (NTU) and NTU Hospital, Taipei, Taiwan; ^7^National Institute of Environmental Health Sciences, National Health Research Institutes, Miaoli County, Taiwan; ^8^Department of Psychiatry, National Taiwan University Hospital, National Taiwan University, Taipei, Taiwan; ^9^Department of Environmental Health, Harvard T.H. Chan School of Public Health, Boston, MA, United States

**Keywords:** greenness exposure, Normalized Difference Vegetation Index, suicide mortality, global analysis, ecological study

## Abstract

**Objective:**

This study applied an ecological-based analysis aimed to evaluate on a global scale the association between greenness exposure and suicide mortality.

**Methods:**

Suicide mortality data provided by the Institute for Health Metrics and Evaluation and the Normalized Difference Vegetation Index (NDVI) were employed. The generalized additive mixed model was applied to evaluate with an adjustment of covariates the association between greenness and suicide mortality. Sensitivity tests and positive-negative controls also were used to examine less overt insights. Subgroup analyses were then conducted to investigate the effects of greenness on suicide mortality among various conditions.

**Results:**

The main finding of this study indicates a negative association between greenness exposure and suicide mortality, as greenness significantly decreases the risk of suicide mortality per interquartile unit increment of NDVI (relative risk = 0.69, 95%CI: 0.59–0.81). Further, sensitivity analyses confirmed the robustness of the findings. Subgroup analyses also showed a significant negative association between greenness and suicide mortality for various stratified factors, such as sex, various income levels, urbanization levels, etc.

**Conclusions:**

Greenness exposure may contribute to a reduction in suicide mortality. It is recommended that policymakers and communities increase environmental greenness in order to mitigate the global health burden of suicide.

## Introduction

Suicide has become a common occurrence in a modern society. Approximately 2.5% of death in the global population has been attributed to suicide (1). Another estimate made by the Institute for Health Metrics and Evaluation 2018 indicated that nearly 10 people per 100,000 population die from committing suicide. Regarding the influence of demographic factors on suicide, suicide mortality rate in men is twice as high as that in women, with death rates of 13.9 and 6.30 per 100,000 population, respectively (2). Furthermore, a study by Turecki & Brent (3) confirmed that gender is a significant factor in suicidal behavior, with higher rates globally of suicide mortality in males (3).

In addition to the influence of gender on suicide mortality, previous studies have suggested that several risk factors trigger psychosocial stress and, thus, inspire suicide attempts, and these factors include mental disorders, socioeconomic and sociocultural problems, physical conditions, and lifestyle (4, 5). In addition to individual factors, recent studies have indicated that outdoor environmental conditions, such as air pollution exposure, climate change, temperature, and seasonal variation have also been associated with suicide (6, 7). The mechanisms by which environments influence suicide attempts is not fully clear, however a prior study identified that environmental conditions and rapid urbanization likely contribute to suicide mortality (8), indicating the significant effects of environmental conditions on suicide.

A recent study revealed that exposure to greenness is negatively associated with suicide mortality (9). Although a limited number of studies have discussed the mechanisms of the effects of greenness on suicide, the beneficial effects of greenness in reducing the physical (10–12) and psychological burdens (13) have been confirmed. It is worth nothing that a systematic review by James in 2015 explored the potential pathways of greenness on health (14). Regardless, environmental greenness is considered to have a positive effect in reducing psychological health burdens which further could influence suicide risk. For example, environmental greenness can active parasympathetic nervous system activity to recover from stress (15) and to reduce negative thoughts resulting from urban stressors, such as crime, traffic noise, and crowding (16). Additionally, environmental greenness offers an open space for social interaction and social coherence (17).

Although previous studies have marked the beneficial effects of greenness on human health, very few studies have investigated on a global scale the linkage between greenness and suicide mortality. Some studies have only utilized a few years of data and/or unequal timeframes of study data (9, 18). Furthermore, several important covariates such as meteorological factors and health status may not have been considered in those studies. Given the paucity of information on the association between greenness and suicide mortality, this study conducted an ecological-based analysis across countries around the world by using 17 years of country-level data collected from various databases. The findings of this study can be applied for reference in regional planning and land-use management.

## Methods

### Data of Suicide Rates and Greenness

#### Suicide Mortality

**Figure 1:**
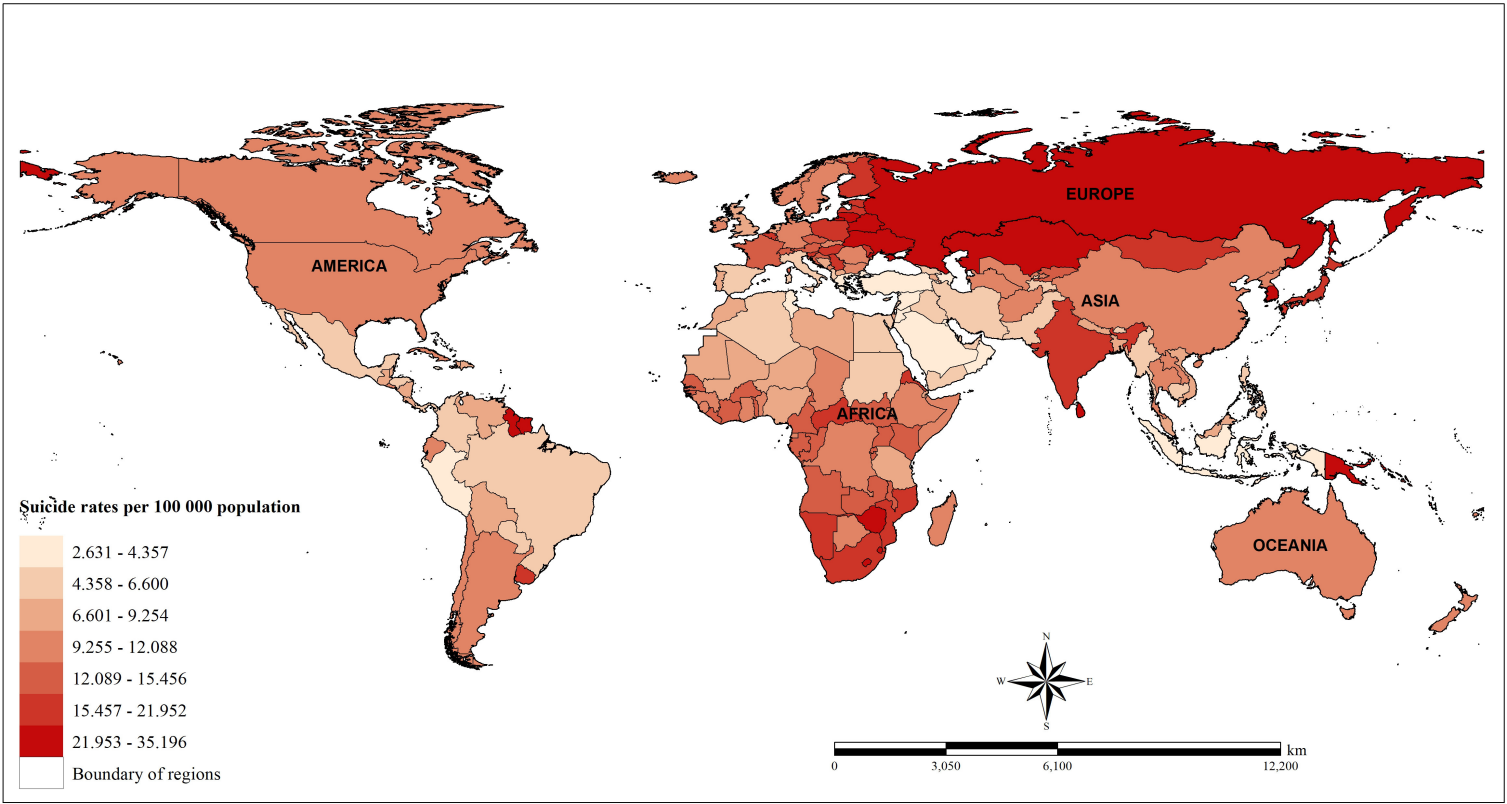
Spatial distribution of suicide rate mortality from 2000 to 2016.

The data of suicide mortality at the country level in this study was provided by the Institute for Health Metrics and Evaluation (IHME) from Global Burden of Disease Collaborative Network—Global Burden of Disease (GBD) study 2017 (http://ghdx.healthdata.org/gbd-results-tool). In this database suicide was defined using the revision codes for intentional injuries (ICD-10 X60-X84, Y870) from the 10th edition of the International Classification of Diseases. Suicide rate mortality was reported by the number of deaths per 100,000 population, which is consistent with the WHO's Indicator and Measurement Registry. Mortality rates were age-adjusted, which is necessary because death rates per 100,000 population are significantly different by age group. Annual estimation data used in this study were available for the seventeen consecutive follow-up years ranging from 2000 to 2016. There were 183 countries across five continents with available data for suicide mortality and related factors. Figure 1 illustrates the spatial distribution of suicide mortality worldwide from 2000 to 2016.

#### Greenness

The Normalized Difference Vegetation Index (NDVI) is a satellite-image-based vegetation index provided by the National Aeronautics and Space Administration (NASA) for estimating plant growth, vegetation cover, and biomass production (19). This estimation is based on chlorophyll from plants, which absorbs visible light for usage in photosynthesis. The algorithm of NDVI produces a range from −1.0 to 1.0, with negative values indicating non-vegetation, such as rock, soil, water, cloud, and ice, and positive values indicating more green vegetation.

**Figure 2:**
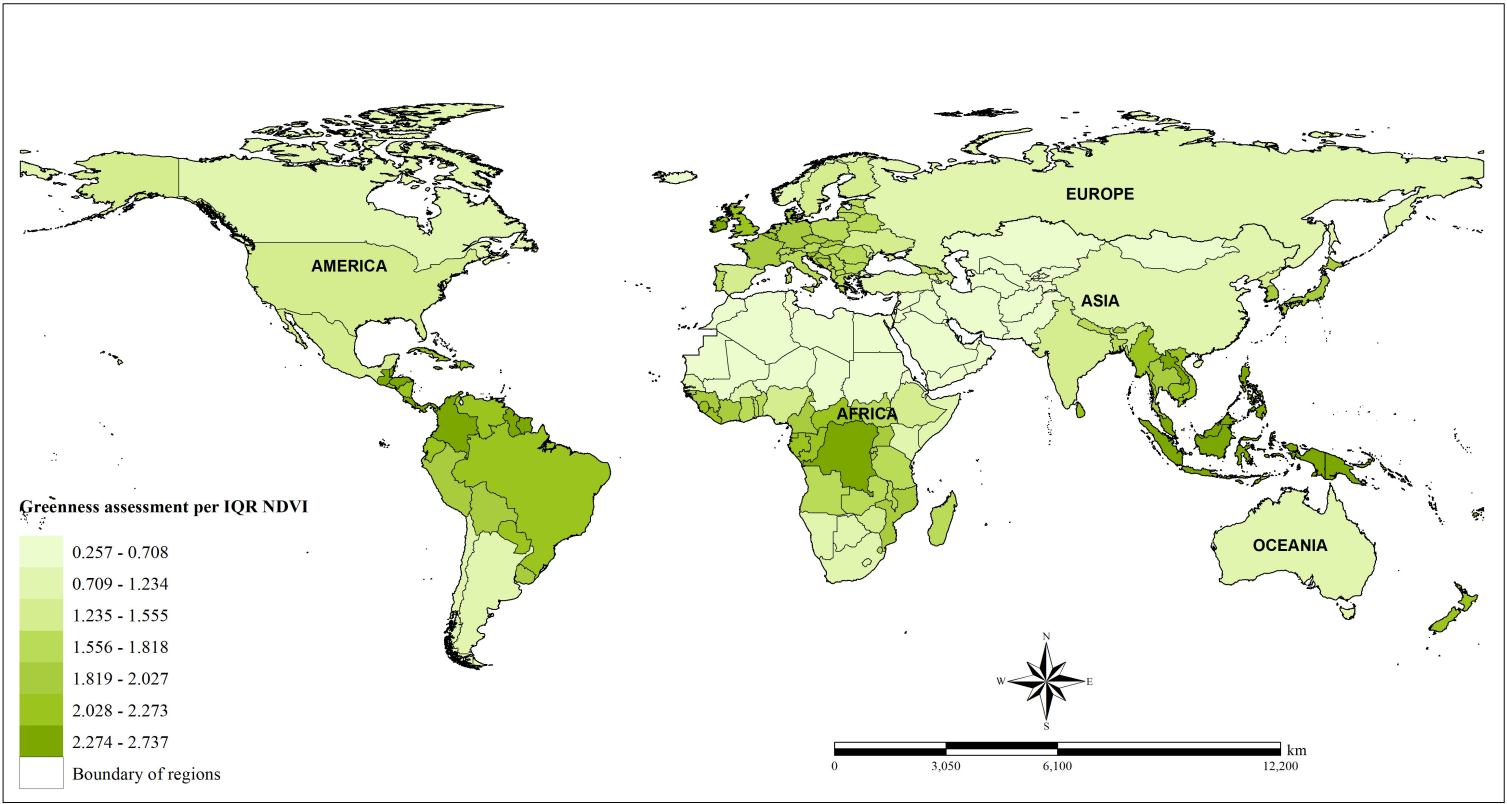
Spatial distribution of greenness measurements (NDVI).

In this study, NDVI data provided by NASA with 1 x 1 km^2^ spatial resolution, as measured by a Terra Moderate Resolution Imaging Spectroradiometer (Terra-MODIS) sensor, were used to estimate greenness exposure in each country (ladsweb.modaps.eosdis.nasa.gov/search/order/1/MOD11A1-6, NASA). The NDVI product used in this study was MOD13A3 version 6. It has been noted the issue of contiguity with water being detected in negative grids of NDVI. Because of this, pixels with negative values were excluded so as to avoid this misclassification bias resulting from the effects of water. Satellite-image NDVI data with acquisition dates closer to the middle of a season were collected for use in this study. In other words, to address seasonal variations, data were retrieved in January, April, July, and October. The month settings in the data collection did account for countries with two and/or four seasons. After that adjustment, a monthly global greenness map was generated by combining 292 images. Moreover, a similar process was performed to estimate the greenness values for each of the four selected months. Finally, monthly greenness estimates were calculated in order to capture the annual average values of greenness at the country level. In total, the number of NDVI images used to generate global greenness maps was 19,856 images (292 x 4 selected months x 17 follow-up years from 2000 to 2016). The spatial distribution of exposure to greenness at the country level based on the interquartile of NDVI (IQR = 0.311) is displayed in Figure 2.

### Potential Covariates

The country-level variables included in this study are listed in Supplementary Table S1 The adjusted covariates were categorized into several groups, including (1) **demographic factors**: density of population, age, and sex (2, 3); (2) **socioeconomic variables**: income level, health expenditure rate, attainment of education rate, urbanization rate, divorce rate, unemployment rate, and population without religious affiliation (20–23); (3) **behavioral factors**: smoking rate and alcohol consumption (24, 25); (4) **air quality and meteorological factors**: air pollution elements, such as temperature and fine particulate matter—PM_2.5_ (26), and temperature (27); (5) **comorbid conditions**: the burden of depressive disorders (DALY loss in years).

### Statistical Modeling and Sensitivity Test

The descriptive statistics of all country-level variables used in this study are presented, and a bivariate test using Spearman's rank algorithm was performed to identify the correlation between suicide mortality and all potential covariates (Supplementary Table S2). The main model, with adjustment for the aforementioned covariates, was completed using the generalized additive mixed model (GAMM) algorithm to investigate the association between greenness and suicide mortality rate. GAMM accounts for both random and fixed effects in calculations and provides a common approach for analyzing health burdens (28). GAMM considered a random intercept with an autoregressive covariance structure, using country ID as the clustered unit, so as to minimize temporal correlation of outcomes resulting from repeated measurements within a country. To control for variability, the Gaussian setting was also considered in the adjustment. Several fixed-effect covariates, such as demographic factors, socioeconomic factors, sociocultural factors, lifestyle behaviors, healthcare status, and mental burdens, were included in the model. The regression spline for centroid longitude-latitude coordinates of the country (fitted degree of freedom = 4) was examined to overcome spatial autocorrelation that occurs when spatial data are included from several different areas (28, 29). The regression spline function also was introduced to control for temperature (fitted degree of freedom = 4). Furthermore, generalized variance-inflation factors (GVIFs) were then tested to examine multicollinearity problems among the covariates (9). Variables with GVIFs <4 were adjusted for in the main model (Supplementary Table S3).

Various sensitivity tests were employed to assess the robustness of the study results. Eight models, each with different covariates, were completed to examine the changes in coefficients and levels of significance. **Model 1** included greenness, density of population, age, sex, healthcare expenditure rate, PM_2.5_, temperature, burden due to depressive disorders, and spatial-temporal autocorrelation; **Model 2** added unemployment rate to model 1; **Model 3** added divorce rate to model 2; **Model 4** added economic status and educational rate to model 3; **Model 5** added urbanization rate to model 4; **Model 6** added the lifestyle behaviors smoking rate and alcohol consumption to model 5; and **Model 7**, we adjusted for all covariates and considering population-weighted greenness as the main exposure. Population-weighted greenness was evaluated considering that exposure to greenness and population density varied spatially in each region (30). In this case, we used the world urban areas dataset which provided the spatial distribution of the global population (31) to adjust greenness estimates. We then adopted the method presented by Chen et al. (27) to calculate the population-weighted greenness (32). Subsequently, positive-negative controls were then introduced as part of the sensitivity analysis in order to validate observed associations between greenness and suicide mortality. For analysis of the positive-negative outcome controls, the association between greenness and the burden of immune disorders (33) were examined as the *positive control*, while the relationship between greenness and total injuries were examined as the *negative control*. Regarding the positive-negative exposure controls, temperature was used as the *positive exposure* (34) while CO_2_ was used as the *negative exposure*.

Subgroup analyses were performed to investigate the association between greenness and suicide mortality for various conditions, and these analyses were stratified by greenness exposure level (low, medium, high exposure), sex (male vs. female), DALY loss due to depressive disorders [< or ≥ median (5.38)], economic status (low, middle, and high-income), urbanization level (low level for urbanization rate <30%; medium level for urbanization rate 30–60%; and high level for urbanization rate > 60%), unemployment rate [< or ≥ median (6.28%)], and divorce rate [< or ≥ median (0.80%)], as well as for level of alcohol consumption [< or ≥ median (2.00 liters/population)] and smoking rate [< or ≥ median (19.10%)].

Each of the statistical analyses were performed using R version 3.6.3 (The R packages Foundation for Statistical Computing, Vienna, Austria) and the spatial analyses were performed using ArcGIS 10.7.1 (Esri Inc., Redlands, California, United States).

## Results

### Descriptive Statistics

The statistical summaries of all variables analyzed in this study are displayed in Tables 1, 2. Over the seventeen-year study period the average suicide mortality rate was 11.27 per 100,000 population [standard deviation (SD) = 6.68]. The median value of NDVI was 1.77 (SD = 0.67), with an IQR of 0.311. Regarding covariates, the average population density for all included countries was 94 persons per km^2^ (SD = 152.72). Nearly half of the total population were aged 25–49 and nearly half were male. Among all of the countries, 80 countries had low-income levels (43.71%). The healthcare expenditure rate, education rate, urbanization rate, divorce rate, unemployment rate, and population without religion were 6.22% (SD = 2.39), 83.7% (SD = 19.68), 54.93% (SD = 22.89), 1.02 (SD = 1.11), 8.23 (SD = 6.61), 8.23 (SD = 6.61), and 0.04 (SD = 0.1), respectively. In terms of behavioral factors, the average smoking rate was 18.6% (SD = 13.79) and the average annual alcohol consumption was 3.26 liters (SD = 3.61). As for meteorological factors, the mean PM_2.5_ exposure was 19.45 μg/m^3^ (SD = 16.10) and the mean temperature was 19.5 °C (SD = 8.16). Lastly, DALY loss due to depressive disorders was 5.62 years (SD = 1.37). The temporal trends of greenness assessment per IQR of NDVI and suicide rates at the country level are illustrated in Supplementary Figure S1. This study identified that, over the course of the 17 years of included data, there were slight changes in both NDVI and suicide mortality.

**Table 1:** Descriptive statistics of continuous variables examined in this study.

**Variable**	**Mean**	**SD**	**Min**	**25th**	**Median**	**75th**	**Max**
**Outcome**						
Suicide rate mortality (people/100,000 population)	11.27	6.68	2.63	6.33	10.19	13.98	35.20
**Exposures**						
Greenness (%, per IQR NDVI)	1.59	0.67	0.26	1.11	1.77	2.10	2.74
**Covariates**						
Density of population (per km^2^)	94.41	152.72	0.54	16.67	48.15	101.05	1327.10
Age 15–49 (yrs, %)	50.65	4.90	40.37	47.30	50.23	53.39	80.33
Age 50–69 (yrs, %)	13.70	6.36	5.46	8.29	11.15	19.34	27.69
Age ≥ 70 (yrs, %)	5.10	3.97	0.39	1.95	3.28	7.75	19.08
Sex (% of male)	50.12	2.30	32.51	49.81	50.39	50.95	54.04
Healthcare expenditure rate (%)	6.22	2.39	0.00	4.62	6.01	7.84	15.88
Education rate (%)	83.70	19.68	0.00	72.60	92.80	98.80	100.00
Urbanization rate (%)	54.93	22.89	8.25	35.44	55.16	73.75	100.00
Divorce rate (%)	1.02	1.11	0.00	0.10	0.80	1.75	5.95
Unemployment rate (%)	8.23	6.61	0.63	3.44	6.28	11.62	35.27
Population without religion (%)	0.04	0.10	0.00	0.00	0.01	0.02	0.77
Smoking rate (%)	18.60	13.79	0.00	6.70	19.10	28.30	73.40
Alcohol consumption (liters/population)	3.26	3.61	0.00	0.21	2.00	4.82	16.64
PM_2.5_ (μg/m^3^)	19.45	16.10	0.46	7.34	15.02	27.45	87.53
Temperature (°C)	19.50	8.16	−7.08	11.88	23.14	25.94	29.54
Burden of depressive disorders (DALY in years)	5.62	1.37	3.04	4.65	5.38	6.47	10.25

**Table 2:** Descriptive statistics of categorical variables examined in this study.

**Variable**	**Number (countries)**	**%**
**Economic status**		
Low-income	80	43.71
Middle-income	52	28.42
High-income	51	27.87

### Association Between Exposure to Greenness and Suicide Mortality

Table 3 lists the relationships between exposure to greenness and suicide mortality. After adjusting for pertinent covariates, the main model identified a significant negative association between suicide mortality and greenness (β = −0.37), with a risk ratio (RR) for suicide of 0.69 (95% CI = 0.81–0.59, *p*-value < 0.001). This result indicates that there is a reduction in the risk of suicide mortality by up to 31% per each unit IQR increment of NDVI (IQR of NDVI = 0.311). Furthermore, the results of the other sensitivity test models demonstrate that greenness exposure is negatively associated with suicide mortality. The results of the analyses demonstrate a robust approximation, which is indicated by the stable estimation of coefficients and corresponding risk ratios. Detailed estimations for all covariates of the main model are presented in Supplementary Table S4.

**Table 3:** Association between greenness and suicide rate mortality.

**Models**	**Coefficient estimation**^**h**^ **(95% CI)**	**Risk ratio**^**h**^ **(95% CI)**	***p*-value**
**Main Model** ^**a**^	**−0.374 (−0.534, −0.213)**	**0.688 (0.586, 0.808)**	**< 0.001**
**Sensitivity test adjusted by covariates**			
**Model 1** ^**b**^	−0.370 (−0.531, −0.210)	0.691 (0.588, 0.811)	<0.001
**Model 2** ^**c**^	−0.370 (−0.524, −0.204)	0.691 (0.588, 0.811)	<0.001
**Model 3** ^**d**^	−0.369 (−0.524, −0.204)	0.690 (0.588, 0.811)	<0.001
**Model 4** ^**e**^	−0.367 (−0.522, −0.201)	0.692 (0.590, 0.813)	<0.001
**Model 5** ^**f**^	−0.370 (−0.525, −0.204)	0.691 (0.588, 0.811)	<0.001
**Model 6** ^**g**^	−0.375 (−0.530, −0.209)	0.688 (0.585, 0.807)	<0.001
**Model 7** ^**h**^	−0.050 (−0.086, −0.013)	0.951 (0.917, 0.986)	<0.01

*CI, Confidence Interval*.

*^a^Adjusted confounder variables included population density, age, sex, burden of depressive disorders, healthcare expenditure, unemployment rate, divorce rate, economic status, educational attainment, urbanization, smoking prevalence, alcohol consumption, population without religion, temperature, PM_2.5_, and spatial –temporal autocorrelation*.

*^b^Adjusted for population density, age, sex, healthcare expenditure, PM_2.5_, temperature, burden of depressive disorders, and spatial –temporal autocorrelation*.

*^c^Adjusted for population density, age, sex, healthcare expenditure, PM_2.5_, temperature, burden of depressive disorders, spatial –temporal autocorrelation, and unemployment rate*.

*^d^Adjusted for population density, age, sex, healthcare expenditure, PM_2.5_, temperature, burden of depressive disorders, spatial –temporal autocorrelation, unemployment rate, and divorce rate*.

*^e^Adjusted for population density, age, sex, healthcare expenditure, PM_2.5_, temperature, burden of depressive disorders, spatial –temporal autocorrelation, unemployment rate, divorce rate, economic status, and educational attainment*.

*^f^Adjusted for population density, age, sex, healthcare expenditure, PM_2.5_, temperature, burden of depressive disorders, spatial –temporal autocorrelation, unemployment rate, divorce rate, economic status, educational attainment, and urbanization*.

*^g^Adjusted for population density, age, sex, healthcare expenditure, PM_2.5_, temperature, burden of depressive disorders, spatial –temporal autocorrelation, unemployment rate, divorce rate, economic status, educational attainment, urbanization, smoking prevalence, and alcohol consumption*.

*^h^Adjusted for all covariates and considering population –weighted greenness as the main exposure (IQR = 0.160)*.

*Per unit IQR of NDVI in the main analysis = 0.311 (31.1%)*.

### Positive-Negative Outcomes and Exposure Control Analysis

Supplementary Table S5 lists the results of the observed associations using positive and negative outcomes and exposure controls. A significant association was observed between greenness and burden due to immune disorders, a positive outcome control. In contrast, the association between greenness and total injuries, a negative outcome control, was found to have no significance. For the positive-negative exposure control analysis, a marginally significant positive association between temperature, a positive exposure control, and suicide mortality was identified. Meanwhile, the association between suicide and CO_2_, a negative exposure control, was found to have no significance.

### Subgroup Analyses

**Figure 3:**
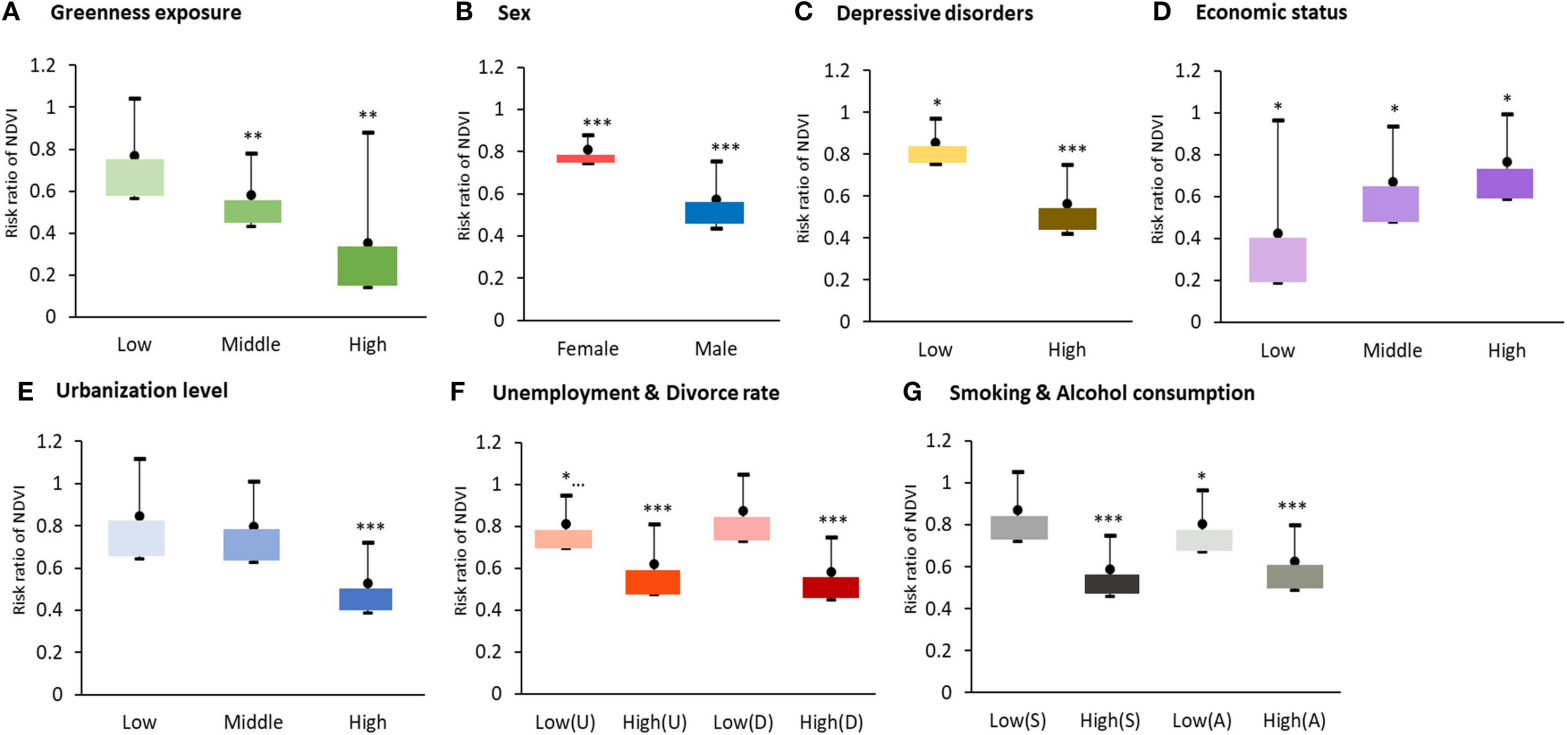
Subgroup analysis among different levels of **(A)** greenness, **(B)** sex, **(C)** burden of depressive disorders, **(D)** economic status, **(E)** urbanization level, **(F)** unemployment rate and divorce rate, **(G)** smoking rate and alcohol consumption in suicide changes per one interquartile unit increment of greenness (NDVI = 0.311). Adjusted variables, including population density, age, sex, burden of depressive disorders, healthcare expenditure rate, unemployment rate (U), divorce rate (D), economic status, educational rate, urbanization rate, smoking rate (S), alcohol consumption (A), population without religion, temperature, PM_2.5_, and spatial-temporal autocorrelation. *p < 0.05, **p < 0.01, ***p < 0.001.

Results of subgroup analyses by different levels of greenness exposure, sex, DALY loss due to depressive disorders, economic status, urbanization rate, unemployment rate, and divorce rate, smoking prevalence, and alcohol consumption are displayed in Figure 3. Regarding greenness exposure, the results indicate significant negative associations between NDVI and suicide mortality at medium and high exposure levels, with RRs of 0.58 and 0.35 (95% CI = 0.43–0.78 and 0.14–0.88, *p*-values < 0.01), respectively. In contrast, no significant association was exhibited between NDVI and suicide mortality in countries with low greenness exposure levels. In terms of sex, significant negative associations were observed for both males and females (male; RR = 0.57, 95% CI = 0.44–0.76; female; RR = 0.81; 95% CI = 0.75–0.88), indicating there is no difference according to sex in relation to greenness in reducing suicide mortality. Additionally, significant negative associations between greenness and suicide mortality were also identified in depressive disorder prevalence (low, RR = 0.86, 95% CI = 0.75–0.97; high, RR = 0.56, 95% CI = 0.42–0.75) and income level (low income, RR = 0.42, 95% CI = 0.19–0.97; middle income, RR = 0.67, 95% CI = 0.48–0.94; high income, RR = 0.77, 95% CI = 0.59–0.99). Significant negative associations between greenness and suicide mortality were observed in countries with the highest unemployment rate (RR = 0.62, 95% CI = 0.47–0.81) and divorce rate (RR = 0.58, 95% CI = 0.45–0.75), as well as in countries with high amounts of alcohol consumption (RR = 0.62; 95% CI = 0.49–0.80) and high smoking rate (RR = 0.59, 95% CI = 0.46–0.75).

## Discussion

This study demonstrated the negative relationship on a global scale between greenness exposure and suicide mortality. This result indicates that exposure to greenness may serve a role in reducing suicide risk. The results of a series of sensitivity analyses were consistent, which bolsters the robustness of the findings, even after adjustment for demographic factors, healthcare status, social environment, and behavioral variables. To our knowledge, this is one of the few ecological studies to have investigated the linkage globally between greenness and suicide mortality. Because they contain analysis of long-term data sets (17 years of data) with comprehensive variables, the study findings provide a global overview of the relationship between greenness and suicide mortality and can be used as a reference in regional planning supporting human health and well-being.

After adjusting for all covariates, this study confirmed the protective effects of greenness on suicide mortality, with a 31% decrease in risk of perceived suicide mortality per IQR increment in greenness. The finding of this large-scale study is consistent with the conclusions drawn in previous studies that have focused on a similar topic but at a smaller geographic level (9, 18). For example, the findings of Helbich's study (2018), drawn from an ecological-based analysis in the Netherlands, confirmed that areas with a medium or high proportion of green space have lower suicide mortality, with relative risks of 0.919 and 0.879, respectively. Our finding is also in line with both the psycho-evolutionary theory and the stress reduction theory, which indicates that connection with nature may evoke positive responses, which in turn promotes a reduction in physiological activation and a blockage of negative thoughts (35). Holtan and colleagues also speculated that public green spaces are open spaces with relatively low-cost and easy interventions to increase the strength of social capital among communities and may could reduce the suicide mortality risk (36). In addition, an environmental theory of suicide proposed by Jiang demonstrated a significant relationship between built environment and suicide mortality (37).

Our stratified analyses revealed the linkage in various conditions between greenness and suicide mortality. In terms of levels of greenness, countries with medium or high greenness exposure levels have a lower suicide mortality rate. These findings corroborate those of previous studies exploring either suicide risk reduction (9) and also other psychological burdens (38). A prior observational study reported that medium greenness exposure levels reduce risks of general health outcomes by up to 2.6% (39). Accordingly, it is suggested that a higher percentage of greenness should be kept and protected for the purpose of health of the population. The findings of this study reflect that greenness exposure has a stronger effect on reducing suicide mortality in countries with high urbanization levels than in countries with low urbanization levels, highlighting the importance of greenness in an ever-urbanizing world. An ecological study in Japan confirmed that green space was significantly associated with a decrease in suicide mortality and the protective effect was higher in densely populated areas (40). Further, investigations conducted in Korea showed similar results, where compared with people living in the high green exposure areas, those living in the lowest green areas had a 16–27% greater chance of experiencing depression and suicide signs (41). This finding also aligns with prior studies that have suggested different urbanization rates affect suicide mortality differently (8). Therefore, seeking a balance between urbanization and nature is vital for urban planning and development. Given the various stressors in urbanized areas, one method to reduce stress and to calm nerves (15), which ultimately will reduce health burdens, is to offer natural landscapes in an open setting.

Regarding prevalence of depressive disorders, our findings support the theory that countries with higher burden from depressive disorders benefit more from greenness exposure's influence on suicide mortality than do countries with lower burden from depressive disorders. It is well known that depression is closely associated with suicidal thoughts and suicide attempts (42). Observational studies in the U.K. have reported that higher levels of green space are linked with lower levels of self-perceived stress due to loneliness (43), as well as lower levels of stress in disparate subgroups, such as the population who are unemployed. This study assessed the impact of greenness exposure on mitigating suicide mortality in countries with distinct psycho-social loads and the results reflect that the beneficial effects of greenness on suicide mortality are significant in countries with the highest rates of divorce and unemployment. Moreover, although this study demonstrates that countries with low-income levels have a greater relative reduction in suicide mortality than countries with medium or high-income levels as an effect of greenness exposure. Furthermore, since the significance of the relationship between greenness exposure and suicide reduction is also found in middle and high-income countries, we argue that countries with higher levels of green are likely to have sufficient investment in the provision of public infrastructure, public services, and facilities. For this reason, the low suicide mortality rate may also be supported by higher levels of social welfare. Regarding health-related behaviors, this study found that exposure to greenness has a significant negative relationship with suicide mortality in countries with a higher prevalence of unhealthy behaviors such as drinking and smoking. This finding is consistent with that of previous studies (44), that the presence of greenness reduces addictive compulsions and cravings, such as those experienced when routinely smoking cigarettes and drinking alcohol, although the mechanism for this is not yet known.

This study has some limitations. *First*, due to a lack of available vegetation data for each country, this study assumed that all types of greenery have a positive impact on health, and the biodiversity of green plants was not considered in the model adjustment. Further investigation is appropriate if detailed information is made available. *Second*, in its analytical models this study controlled for many pertinent factors, but several confounding covariates may not have been considered, such as individual health information, genetics, family history, hereditary diseases, race/ethnicity, and cultural tolerance. Because of this, possible unmeasured confounding effects remain unknown. *Third*, a country-level database may not be the best scale for variable assessments, and it may introduce statistical bias. However, due to the limited availability of city-level data for all selected variables, we chose to use country-level data to examine the global association between greenness and reduced risk of suicide mortality. *Fourth*, MODIS-NDVI with 1 x 1 km^2^ resolution was used for the greenness assessment. We recognize that 1 x 1 km^2^ is not the optimal spatial resolution of MODIS-NDVI for estimating greenness and could not distinguish the type of green spaces. Thus, the use of MODIS-NDVI with the best spatial resolution of 250 x 250 m^2^ for future studies is suggested. In addition, we also understand that this study lacks land use or land cover data that may be more reliable to assess exposure to urban green spaces including public and private green spaces. *Fifth*, although global suicide data has gone through a process of standardization and evaluation of data quality using sophisticated modeling frameworks, however, data quality gaps may exist in each country. This may be due to under-reporting of suicide as a social, cultural, religious, and political or legal consequence, especially in lower-middle-income countries. Finally, we recognize that global analysis using country-level data have shortcomings in terms of data accuracy and result interpretation. Therefore, to support better scientific evidence, use of accurate data and comprehensive analysis is recommended for future studies.

Notwithstanding the shortcomings of this study, our findings could serve to help better understand at a macro level the relationship between greenness exposure and suicide mortality. A series of sensitivity tests were performed in order to confirm the robustness of the findings. More detailed information was obtained from the ensuing subgroup analyses. Based on this information, the study proposed suggestions to and offered encouragement to decision-makers, key stakeholders, and community members to heed calls for a spatial planning that supports place-based suicide prevention programs.

## Conclusion

This study serves a pivotal role in a better understanding of how exposure to greenness can be linked to a reduced risk of suicide mortality globally. The observed reduced effect of suicide mortality was related to greenness exposure under various conditions. Confessing interventions from exposure to greenness can affect general health outcomes in the extensive population, this study also proposed suggestion and encouragement to the entire community and stakeholders to pay attention to spatial planning that supports place-based suicide prevention programs.

## Data Availability Statement

The data set from this study is publicly available. Data used in this study was acquired from the Institute for Health Metrics and Evaluation - IHME, which provided the metrics of disability-adjusted life years (DALY) database (ghdx.healthdata.org/gbd-results-tool, Accessed in November 2020); the National Aeronautics and Space Administration - NASA, which provided global greenness NDVI data (ladsweb.modaps.eosdis.nasa.gov/search/order/1/MOD11A1-6, Accessed in November 2020); the Atmospheric Composition Analysis Group, which provided global PM2.5 data (fizz.phys.dal.ca/~atmos/martin/?page_id=140, Accessed in May 2020); the United Nations Agency, which provided demographic data (esa.un.org/unpd/wpp/); and the World Bank Group, which provided data for socioeconomic status, smoking prevalence, alcohol consumption, and pertinent covariates at the country level (data.worldbank.org/indicator/SE.ADT.LITR.ZS; apps.who.int/gho/data/node.main.A1039?lang=en; data.un.org/Data.aspx?q=religion&d=POP&f=tableCode%3a28).

## Author Contributions

AKA, H-JS, C-DW, and JDS: conceptualization. AKA, H-JT, W-CP, Y-LG, and C-DW: methodology. AKA and H-JT: formal analysis. AKA, H-JT, and C-DW: writing—original draft preparation. AKA, H-JT, P-YW, H-YL, W-CP, Y-LG, C-SW, H-JS, C-DW, and JDS: writing—review and editing. C-DW and H-JS: supervision and funding acquisition. All authors contributed to the article and approved the submitted version.

## Funding

This study was funded in part by the Ministry of Science and Technology, R.O.C. (MOST 110-2628-M-006-001-MY3).

## Conflict of Interest

The authors declare that the research was conducted in the absence of any commercial or financial relationships that could be construed as a potential conflict of interest.

## Publisher's Note

All claims expressed in this article are solely those of the authors and do not necessarily represent those of their affiliated organizations, or those of the publisher, the editors and the reviewers. Any product that may be evaluated in this article, or claim that may be made by its manufacturer, is not guaranteed or endorsed by the publisher.
