# Supplementary material for: Examining the Benefits of Greenness on Reducing Suicide Mortality Rate: A Global Ecological Study
Source: Front Public Health. 2022 Jul 5;10:902480. doi: 10.3389/fpubh.2022.902480 (PMC9294351; doi:10.3389/fpubh.2022.902480)

Table S1. Detailed information of covariates

Covariate	Description	Data Source	Year of data
Demographic factors (population density, age, sex)	We used country-level demographics data including density of population per km^2, age, and sex. In line with the suicide rate database, we considered proportion values from three age groups above 15 years old in the analysis (15-49, 50-69, and >70 years). Sex was represented by percentage of male; the male has a higher risk of suicide than female (IHME, 2018)	United Nations, Department of Economic and Social Affairs.	2000 - 2016
Economic status	Three levels of economic status (i.e., low-income, middle-income, high-income countries) were used in this study. Classification of economic status of each country was based on the 2016 Atlas gross national income per capita.	World Bank Group	2000 - 2016
Healthcare expenditure rate	Total health expenditure including the provision of health services (preventive and curative), family planning activities, nutrition activities, and emergency aid designated for health were taken into account in the adjustment.	World Bank Group	2000-2014
Education rate	We analyzed the prevalence rate of education on a country-level.	World Bank Group	2000 - 2016
Urbanization rate	We used the prevalence rate of the urban population in each country.	World Bank Group	2000 - 2016
Divorce rate	We used the prevalence of divorce data in each country.	United Nations, Department of Economic and Social Affairs	2008

Covariate	Description	Data Source	Year of data
Unemployment rate	We considered the prevalence rate of unemployment on a country-level.	World Bank Group	2000 - 2016
Population without religion	We collected data to find out the prevalence rates of the population without religions in each country.	United Nations Statistics Division	2000 - 2016
Smoking rate	We used the prevalence rate of smoking data at the country- level.	World Bank Group	2000 - 2016
Alcohol consumption	We used the average number of alcohol consumption in liters per population.	World Bank Group	2000 - 2016
$PM_{2.5}$	We estimated $PM_{2.5}$ concentration variations on a global scale with spatial resolution 1x1 km. $PM_{2.5}$ data is a daily total column of aerosol optical depth retrievals from satellites was coupled with the GEOS-Chem transport model and geographically weighted regression model (van Donkelaar et al. 2016).	The Atmosphere Composition Analysis Group established by Prof. Randall Martin from Dalhousie University.	2000 - 2016
Temperature	We obtained high-resolution grids of monthly temperature data and then we calculated annual average temperature. This dataset is produced by the Climatic Research Unit of the University of East Anglia (UEA).	The Climate Change Knowledge Portal	2000 - 2016
Burden of depressive disorders	We considered the burden of depressive disorders (ICD-10, F32-F33, F34.1) as covariates and represented by the disability-adjusted life year (DALY loss) estimations.	World Health Organization	2000 - 2016

Table S2. Correlation assessment of suicide mortality and pertinent covariates using Spearman algorithm.

Variables	*ρ*
**Exposure**	
Greenness (NDVI)	-0.116**
**Covariates**	
Density of population	-0.078**
Age 15 - 49 (%)	-0.196**
Age 50 - 69 (%)	0.112**
Age ≥ 70 (%)	0.118**
Sex (male %)	0.291**
Economic status	-0.061**
Healthcare expenditure rate (%)	-0.104**
Education rate (%)	-0.009
Urbanization rate (%)	-0.155**
Divorce rate (%)	0.132**
Unemployment rate (%)	0.068**
Population without religion (%)	0.079**
Smoking rate (%)	0.195**
Alcohol consumption (liters/population)	0.169**
$PM_{2.5}$ ($\mu g/m^3$)	0.023
Temperature (ºC)	0.121*
Burden of depressive disorders	0.234**

* *p-value <0.05;* ** *p-value <0.01;* *** *p-value <0.001*

Table S3. The result of multicollinearity test

Variables	GVIFs
**Exposure**	
Greenness (NDVI)	1.348
**Covariates**	
Density of population	1.080
Age 15 - 49 (%)	1.730
Age 50 - 69 (%)	3.335
Age ≥ 70 (%)	3.804
Sex (male %)	1.515
Economic status	2.097
Healthcare expenditure rate (%)	1.223
Education rate (%)	1.851
Urbanization rate (%)	1.501
Divorce rate (%)	1.459
Unemployment rate (%)	1.101
Population without religion (%)	1.049
Smoking rate (%)	1.217
Alcohol consumption (liters/population)	1.362
$PM_{2.5}$ (μg/m^3)	1.036
Temperature (ºC)	1.761
Burden of depressive disorders (DALY in year)	1.269

Note. Variables with GVIFs > 4 thought to be problematic and could make the model biased.

Table S4. Estimation of main model

Variables	Coefficient estimation [a] (95% CI)	RR of variable [a] (95% CI)
Greenness (NDVI)	-0.374 (-0.534, -0.213) ***	0.688(0.586, 0.808) ***
Burden of depressive disorders (DALY in year)	0.005 (0.003, 0.006) ***	1.005 (1.003, 1.007) ***
Healthcare expenditure rate (%)	-0.017 (-0.038, -0.004)	0.983 (0.963, 1.000)
Density of population (per km^2)	-0.001 (-0.002, -0.000) *	0.999 (0.998, 0.999) *
Sex (% of male)	0.011 (-0.052, 0.075)	1.012 (0.950, 1.078)
Age 15 - 49 (%)	-0.022 (-0.046, 0.002)	0.978 (0.955, 1.002)
Age 50 - 69 (%)	-0.035 (-0.067, -0.003) *	0.966 (0.935, 0.997) *
Age ≥ 70 (%)	-0.082 (-0.150, -0.015) *	0.920 (0.861, 0.985) *
Economic status		
Low-income	*Reference*	
Middle-income	-1.132 (-3.603, 1.339)	-
High-income	-1.553 (-4.304, 1.198)	-
Urbanization rate (%)	-0.014 (-0.027, 0.001) *	0.986 (0.973, 0.999) *
Education rate (%)	-0.049 (-0.113, 0.014)	0.952 (0.893, 1.015)
Unemployment rate (%)	0.009 (-0.016, 0.020)	1.002 (0.985, 1.020)
Population without religion (%)	0.005 (-0.002, 0.012)	1.005 (0.998, 1.012)
Divorce rate (%)	0.009 (0.000, 0.019) *	1.009 (1.000, 1.020) *
Alcohol consumption (liters/population)	0.017 (0.000, 0.034) *	1.017 (1.000, 1.034) *
Smoking rate (%)	0.008 (-0.005, 0.020)	1.008 (0.996, 1.021)

Abbreviations: CI= Confidence Interval, RR = Risk Ratio

* *p-value* <0.05; ** *p-value* <0.01; *** *p-value* <0.001

[a.] Confounder variables included population density, age, sex (% of male), burden of depressive disorders, healthcare expenditure, unemployment, divorce rates, economic status, the prevalence rate of education, urbanization, the prevalence rate of smoking, alcohol consumption, population without religion, temperature, $PM_{2.5}$, year, and spatial-temporal autocorrelation.

Table S5. Positive-negative outcome and exposure controls analysis

Positive – negative outcome controls						
	**Greenness related to immune disorders** (positive)			**Greenness related to total injuries** (negative)		
	**Coefficient** (95% CI)	**RR** (95% CI)	**p-value**	**Coefficient** (95% CI)	**RR** (95% CI)	**p-value**
**Model 1** [a]	-0.126 (-0.230, -0.023)	0.881 (0.795,0.978)	<0.05	0.144 (-0.097, 0.384)	1.155 (0.908,1.469)	0.24
**Model 2** [b]	-0.126 (-0.231, -0.022)	0.881 (0.794,0.978)	<0.05	0.110 (-0.131, 0.123)	1.116 (0.877,1.420)	0.37
**Positive – negative exposure controls**						
	**Temperature related suicide rates** (positive)			**CO_2 related to suicide rates** (negative)		
	**Coefficient** (95% CI)	**RR** (95% CI)	**p-value**	**Coefficient** (95% CI)	**RR** (95% CI)	**p-value**
**Model 1** [a]	0.033 (-0.004, 0.071)	1.034 (0.996, 1.072)	0.07	-0.041 (-0.186, 0.104)	0.960 (0.830,1.110)	0.58
**Model 2** [c]	0.034 (-0.003, 0.070)	1.034 (0.997, 1.073)	0.08	-0.101 (-0.270, 0.068)	0.904 (0.763,1.071)	0.24

Abbreviation: CI= Confidence Interval, RR = Risk Ratio

[a.] Adjusted for population density, sex (% of male), age, $PM_{2.5}$, temperature, and spatial-temporal autocorrelation.

[b.] Control variables included population density, sex (% of male), age, economic status (level of income), the prevalence rate of smoking, alcohol consumption, the prevalence rate of education, urbanization, $PM_{2.5}$ exposures, and spatial-temporal autocorrelation.

[c.] Control variables included population density, sex (% of male), burden of depressive disorders, healthcare expenditure rate, unemployment rate, divorce rate, economic status, the prevalence rate of education, urbanization, the prevalence rate of smoking, alcohol consumption, population without religion, temperature, $PM_{2.5}$, and spatial-temporal autocorrelation.

Figure S1. Temporal trends of Global greenness measurements and Global suicide rate mortality from 2000 to 2016

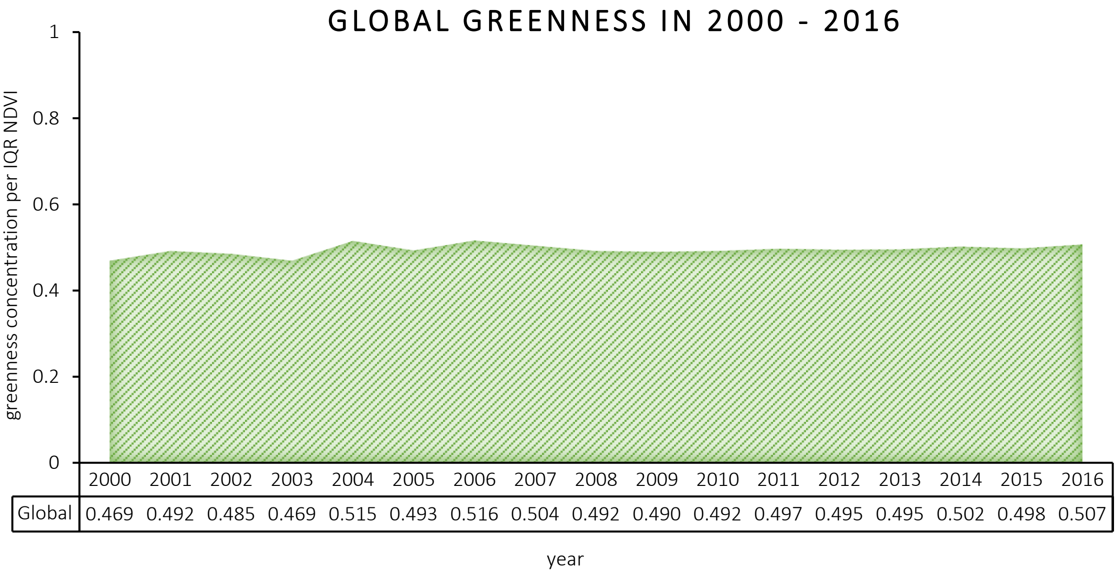

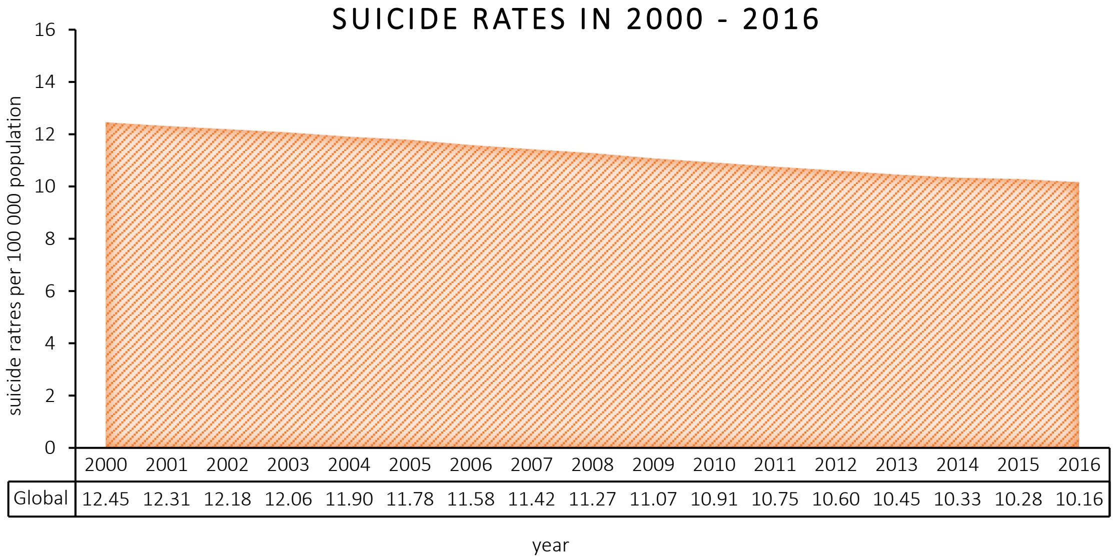

Supplement: Supplementary file 1 [file Data_Sheet_1.pdf]
